# Personalized assessment of the cumulative complication risk of the atrial fibrillation ablation track: The AF-TRACK calculator

**DOI:** 10.1016/j.hroo.2022.07.013

**Published:** 2022-08-09

**Authors:** Felipe Bisbal, Juan-Pablo Abugattas, Omar Trotta, Juan José Gonzalez-Ferrer, Assumpció Sauri-Ortiz, Miguel Angel Arias, Isaac Subirana, Mattias Duytshaever, Jan De Pooter, Axel Sarrias, Raquel Adeliño, Francisco Alarcón, Lluís Mont, Julián Pérez-Villacastín, Joaquin Osca-Asensi, Roger Villuendas, Marta Pachón-Iglesias, Milad El Haddad, Antoni Bayés-Genís, Yves de Greef

**Affiliations:** ∗ Heart Institute–Hospital Universitari Germans Trias i Pujol, Badalona, Spain; † Cardiovascular Disease Networking Biomedical Research Center (CIBERCV), Spain; ‡ ZNA Heart Center, Middelheim, Antwerpen, Belgium; § Hospital Clinic de Barcelona, Barcelona, Spain; ¶ Hospital Clínico San Carlos, Madrid, Spain; ‖ Hospital La Fe de Valencia, Valencia, Spain; ∗∗ Hospital Virgen de la Salud, Toledo, Spain; †† UZ Ghent, Ghent, Belgium; ‡‡ Sint-Jan Hospital Bruges, Belgium; §§ Heart Rhythm Management Centre, Universitair Ziekenhuis Brussel, Vrije Universiteit Brussel, Brussels, Belgium

**Keywords:** Ablation, Atrial fibrillation, Complication, Prediction, Risk assessment

## Abstract

**Background:**

Atrial fibrillation (AF) ablation strategy is associated with a non-negligible risk of complications and often requires repeat procedures (AF ablation track), implying repetitive exposure to procedural risk.

**Objective:**

The purpose of this study was to develop and validate a model to estimate individualized cumulative risk of complications in patients undergoing the AF ablation track (Atrial Fibrillation TRAck Complication risK [AF-TRACK] calculator).

**Methods:**

The model was derived from a multicenter cohort including 3762 AF ablation procedures in 2943 patients. A first regression model was fitted to predict the propensity for repeat ablation. The AF-TRACK calculator computed the risk of AF ablation track complications, considering the propensity for repeat ablation. Internal (cross-validation) and external (independent cohort) validation were assessed for discrimination capacity (area under the curve [AUC]) and goodness of fit (Hosmer-Lemeshow [HL] test).

**Results:**

Complications (N = 111) occurred in 3.7% of patients (2.9% of procedures). Predictors included female sex, heart failure, sleep apnea syndrome, and repeat procedures. The model showed fair discrimination capacity to predict complications (AUC 0.61 [0.55–0.67]) and likelihood of repeat procedure (AUC 0.62 [0.60–0.64]), with good calibration (HL χ^2^ 12.5; *P* = .13). The model maintained adequate discrimination capacity (AUC 0.67 [0.57–0.77]) and calibration (HL χ^2^ 5.6; *P* = .23) in the external validation cohort. The validated model was used to create the Web-based AF-TRACK calculator.

**Conclusion:**

The proposed risk model provides individualized estimates of the cumulative risk of complications of undergoing the AF ablation track. The AF-TRACK calculator is a validated, easy-to-use, Web-based clinical tool to calibrate the risk-to-benefit ratio of this treatment strategy.

**Key Findings**

▪ The invasive rhythm control strategy for treatment of atrial fibrillation (AF) is not limited to a single procedure and often requires repeat interventions (AF ablation track) to achieve rhythm and symptoms control and hence implies a repetitive exposure to procedural risk.
▪ The proposed risk prediction model provides individualized estimates of the propensity for repeat ablation and the risk of major complications of the AF ablation track.
▪ The Atrial Fibrillation TRAck Complication risK (AF-TRACK) calculator offers a validated, easy-to-use, Web-based clinical tool that allows clinicians and patients with AF to better calibrate the risk-to-benefit ratio of undertaking an invasive treatment strategy.

## Introduction

Atrial fibrillation (AF), the most common arrhythmia, has a marked impact on quality of life, morbidity, and mortality, and is considered a world epidemic.^1^^,^^2^ In the subgroup of patients with symptoms of AF, ablation has become the cornerstone to achieving rhythm control when medical treatment is ineffective. Since the first description in 1998 by Haissaguerre, the number of AF ablation procedures has grown exponentially (annual growth up to 30%), becoming the most common ablation procedure in developed countries.^3^, ^4^, ^5^ AF ablation is generally considered a safe procedure, with a reported 5%–6% rate of major complications^6^ but ranging from 0.9%–13.8%, a difference mostly due to poor agreement in definitions and reporting across studies.^7^^,^^8^ Despite technical advances and improved operator experience, the single-procedure success rate remains moderate, especially in patients with persistent and long-standing persistent AF. Thus, the ablation strategy is not limited to a single procedure and requires repeat interventions (AF ablation track) in 15%–40% of cases to achieve symptomatic relief and rhythm control,^4^ which implies a repetitive exposure to procedural risk and increases cumulative risk.^9^

The aim of the study was to develop and validate a risk prediction model of complications and the propensity of repeat interventions, based on readily available clinical predictors, to provide individualized, preprocedural estimates of cumulative risk of complications from undergoing an AF ablation track (AAT).

## Methods

### Study population

The derivation dataset used to generate the prediction model was obtained from a multicenter cohort of unselected consecutive patients undergoing *de novo* or repeat ablation of symptomatic drug-refractory paroxysmal and persistent AF at 6 institutions in Spain and Belgium (Germans Trias i Pujol University Hospital, Badalona, Spain; ZNA Middelheim, Antwerp, Belgium; Hospital Clinic, Barcelona, Spain; Hospital Clínico San Carlos, Madrid, Spain; Hospital Virgen de la Salud, Toledo, Spain; and Hospital La Fe, Valencia, Spain). An independent cohort of unselected consecutive patients undergoing AF ablation was used for external validation (Ghent University Hospital, Ghent, Belgium). The study complies with the Declaration of Helsinki. All patients had given consent for their inclusion in institutional AF ablation databases, and the study protocol was approved by the responsible Ethics Committee. The data underlying this article will be shared on reasonable request to the corresponding author.

### AF ablation track

Ablation procedures were performed according to each institutional protocol and included contact and noncontact force sensing catheters, as well as first- and second-generation cryoballoon technologies. Radiofrequency ablation consisted of a wide antral circumferential lesion set to achieve pulmonary vein isolation (PVI) in all cases. Additional substrate ablation was performed according to physician criteria. Operators reported adding these lesions in selected cases with a very dilated left atrium (LA) or the presence of organized LA tachycardia after PVI. Cryoballoon-based ablation consisted of occlusive cryoballoon energy applications of up to 240 seconds to achieve PVI (bonus freeze was performed generally), confirmed by an inner-lumen spiral mapping catheter. Cryoenergy at right-side pulmonary veins was applied under phrenic nerve monitoring by pacing at the superior vena cava. In a small proportion of procedures, other catheter-based technologies were used and included pulmonary vein ablation catheter (PVAC), laser balloon, and high-density mesh ablator.^10^, ^11^, ^12^

All centers reported active involvement of training fellows during the procedure, including transseptal puncture, mapping, and ablation. Five of 6 centers performed the procedure with patients under general anesthesia and transesophageal echocardiographic guidance. Most centers adopted an uninterrupted oral anticoagulation strategy following current clinical guidelines. All patients received postprocedure oral anticoagulation therapy with anti–vitamin K or direct-action oral anticoagulants according to guidelines. In most cases, transesophageal echocardiography was performed before ablation or during the procedure to exclude the presence of LA thrombus. Heparin was administered before or after transseptal puncture, according to center protocol and patient weight, to maintain an activated clotting time of 300–350 seconds.

Repeated procedures were performed in cases of symptomatic AF recurrence, aiming to identify and reisolate the reconnected pulmonary veins. Additional ablation sets were performed in selected patients according to physician criteria.

Patients were routinely hospitalized postprocedure for 24 hours. Patient follow-up included outpatient clinic visits at 3, 6, and 12 months postablation. Patients were instructed to seek medical care whenever they experienced symptoms. Except for 1 center, no systematic imaging of the LA was performed in most cases.

### Complications

Major complications were defined according to the latest consensus document as any complication that results in permanent injury or death, requires intervention for treatment, or prolongs or requires hospitalization for more than 48 hours.^6^ The following complications were considered: death, atrioesophageal fistula, cardiac tamponade, transient ischemic accident or stroke, acute pulmonary edema/heart failure decompensation, symptomatic pulmonary vein stenosis, and persistent phrenic nerve palsy. Heart failure was defined according to the CHA_2_DS_2_-VASc score as a previous episode of decompensated heart failure irrespective of left ventricular (LV) ejection fraction or presence of moderate-to-severe LV systolic dysfunction (LV ejection fraction <50%). Vascular access complications (VACs) included severe hematoma, arterial pseudoaneurysm, and arteriovenous fistula. Prolongation of hospitalization due to atrial arrhythmia recurrence was not considered a complication.

### Statistical analysis

All analysis was performed using R software Version 4.0.3 (R Core Team 2020, R Foundation for Statistical Computing, Vienna, Austria). Categorical variables are given as frequency (percentage). Continuous variables are given as median [interquartile range]. Medians and proportions were compared by Kruskal-Wallis test and χ^2^ test, respectively, or Fisher exact test when expected proportions were <5.

#### Models

A logistic regression model was fitted to compute the probability of undergoing a repeat procedure (≥2 ablations). This probability, also known as propensity, has no direct interpretation and is computed as a tool replacing the proportion of ≥2 procedures because the latter cannot be used in the predictive model for complications. A second logistic regression model was fitted to predict complications using leave-one-out methodology given patient characteristics, such as age and gender, and including the propensity for repeat ablation. The variables included in the 2 models were selected by stepwise procedure under the Aikake information criterion forcing the age to be included in the model. Variables with significant missing data were discarded after testing in a multiple imputation analysis. The same methodology was followed to compute the predictive model for VAC. The equation to compute the probability of suffering a complication was implemented in a friendly Web user interface (Atrial Fibrillation TRAck Complication risK [AF-TRACK] calculator) (R Shiny package tools, https://shiny.rstudio.com/).

#### Validation

Discrimination capacity of computed probabilities to predict complication was assessed by the area under the receiver operating characteristic curve (AUC), goodness-of-fit by Hosmer-Lemeshow (HL) test. In the training set, AUC and HL test were computed using the leave-one-out strategy, a cross-validation strategy that prevents obtaining overly optimistic validation results on the training set (overfitting). In addition, an external validation (AUC and HL test) was performed in an external, independent cohort different from the training dataset.

## Results

### Study population and procedural data of the training cohort

The training cohort included 3046 patients who underwent a total of 3912 procedures. VACs occurred in 103 patients (3.4%): major complications in 46 patients (1.5%) and minor in 57 (1.9%). Patients with major VACs were analyzed in a different model. In total, 2943 patients (3762 procedures) were analyzed. Baseline patient characteristics are detailed in Table 1. Mean age was 58 ± 11 years, 73% were men, and paroxysmal AF was the most common indication for ablation (59%). Radiofrequency was the most common energy source at *de novo* (71%) and repeat (99%) ablation procedures, followed by cryoballoon (14.2%), PVAC (8.6%), laser balloon (3.7%), and high-density mesh ablator (2.5%). PVI was the main procedural endpoint and was achieved in all patients. Additional substrate modification was uncommon at *de novo* ablation but increased in repeat procedures (16.3% vs 21.6%; *P* = .002), with roof and lateral mitral isthmus lines being the most frequently performed (detailed ablation sets are given in Supplementary Table S1). A total of 691 patients (23%) underwent 2 procedures (577) or >2 procedures (114 had 3 procedures and 14 had 4 procedures). Mean length of the AF ablation track was 1.28 procedures per patient. A comparison of patient characteristics according to number of procedures is given in Supplementary Table S2.

**Table 1:** Baseline characteristics of the derivation cohort

		No.
Age (y)	57.6 ± 10.6	3046
Female sex	855 (28)	3046
Hypertension	1358 (44.5)	3046
Diabetes	242 (7.9)	3046
Previous CVA/TIA	129 (4.2)	3046
Body mass index (kg/m^2^)	27.0 ± 4.6	2439
Paroxysmal AF	1801 (59)	3046
Heart failure	167 (5.5)	3046
Coronary artery disease	160 (5.2)	3046
Valvular disease	54 (1.8)	3046
Sleep apnea syndrome	290 (9.5)	2760
CHA_2_DS_2_VASc score	1.3 (1.2)	3046
Left atrial diameter (mm)	42.4 ± 6.2	2530
Left ventricular ejection fraction (%)	59.1 ± 9.3	1680
Procedural data (*de novo*)		
Procedural time (min)	165.3 ± 56.9	2575
Fluoroscopy time (min)	26.7 ± 16.8	2503
Ablation energy source		3046
Radiofrequency	2163 (70.9)	
Cryoballoon	434 (14.2)	
Other	453 (14.9)	
Procedural data (repeat)		
Procedural time (min)	168.0 ± 121.4	326
Fluoroscopy time (min)	23.3 ± 12.2	312
Ablation energy source		862
Radiofrequency	855 (99.2)	
Cryoballoon	4 (0.5)	
Other	3 (0.3)	

Values are given as mean ± SD or n (%).

AF = atrial fibrillation; CHA_2_DS_2_-VA_2_Sc = congestive heart failure, hypertension, age ≥75 years, diabetes mellitus, stroke or transient ischemic attack, vascular disease, age 65 to 74 years, sex category; CVA = cerebrovascular accident; TIA = transient ischemic attack.

### Complications

In total, 111 complications occurred in 110 patients (2.9% of procedures and 3.7% of patients [AAT complication rate]). Neither deaths nor atrioesophageal fistulas were reported in the derivation cohort. Cardiac tamponade was the most common complication (1.2%) (Table 2). Patients undergoing ≥2 ablations had double the rate of complications compared to those undergoing *de novo* ablation alone (6% vs 3%; *P* = .001). A detailed comparison of baseline clinical characteristics according to presence of complications is given in Table 3. Data on patient characteristics by VAC are given in Supplementary Table S3.

**Table 2:** Major complications by procedure type (derivation cohort)

	*De novo*	Repeat	Total
	(n = 2943)	(n = 819)	(n = 3762)
Complications	85 (2.9)	26 (3.2)	111 (3.0)
Death	0 (0)	0 (0)	0 (0)
Atrioesophageal fistula	0 (0)	0 (0)	0 (0)
Heart failure	9 (0.3)	4 (0.5)	13 (0.3)
Cardiac tamponade	40 (1.4)	8 (1.0)	48 (1.3)
Symptomatic pulmonary vein stenosis	4 (0.1)	0 (0)	4 (0.1)
Acute coronary syndrome	8 (0.3)	4 (0.5)	12 (0.3)
Stroke/transient ischemic attack	12 (0.4)	9 (1.1)	21 (0.6)
Phrenic nerve palsy	14 (0.5)	1 (0.1)	15 (0.4)

Values are given as n (%).

**Table 3:** Baseline and procedural characteristics by the presence of complications (derivation cohort)

	No complications	Complications	*P* value
	(n = 2833)	(n = 110)	
No. of procedures			.002
1	2183 (77.1)	69 (62.7)	
2	546 (19.3)	31 (28.2)	
>2	104 (3.67)	10 (9.09)	
Age (y)	58.2 [50.6–65.3]	61.5 [53.2–66.4]	.037
Female sex	764 (27.0)	46 (41.8)	.001
Hypertension	1236 (43.6)	54 (49.1)	.301
Diabetes mellitus	217 (7.66)	12 (10.9)	.286
Body mass index (kg/m^2^)	26.6 [23.7–29.1]	28.0 [25.0–30.8]	.006
Nonparoxysmal AF	1166 (41.2)	43 (39.1)	.739
Heart failure	144 (5.08)	13 (11.8)	.004
Coronary artery disease	148 (5.22)	3 (2.73)	.345
Valvular heart disease	44 (1.55)	4 (3.64)	.102
Sleep apnea syndrome	262 (9.25)	17 (15.5)	.044
Left ventricular ejection fraction (%)	60.0 [55.0–65.0]	60.0 [55.0–65.8]	.548
CHA_2_DS_2_-VA_2_Sc score	1.00 [0.00–2.00]	2.00 [1.00–3.00]	.001
Left atrial diameter (mm)	42.0 [38.0–46.0]	43.0 [40.0–46.0]	.178
Center volume (≥100/y)	2268 (80.1)	79 (71.8)	.047
PVI alone	2336 (82.5)	90 (81.8)	.964

Values are given as n (%) or median [interquartile range] unless otherwise indicated.

PVI = pulmonary vein isolation; other abbreviations as in Table 1.

### Model development

**Figure 1:**
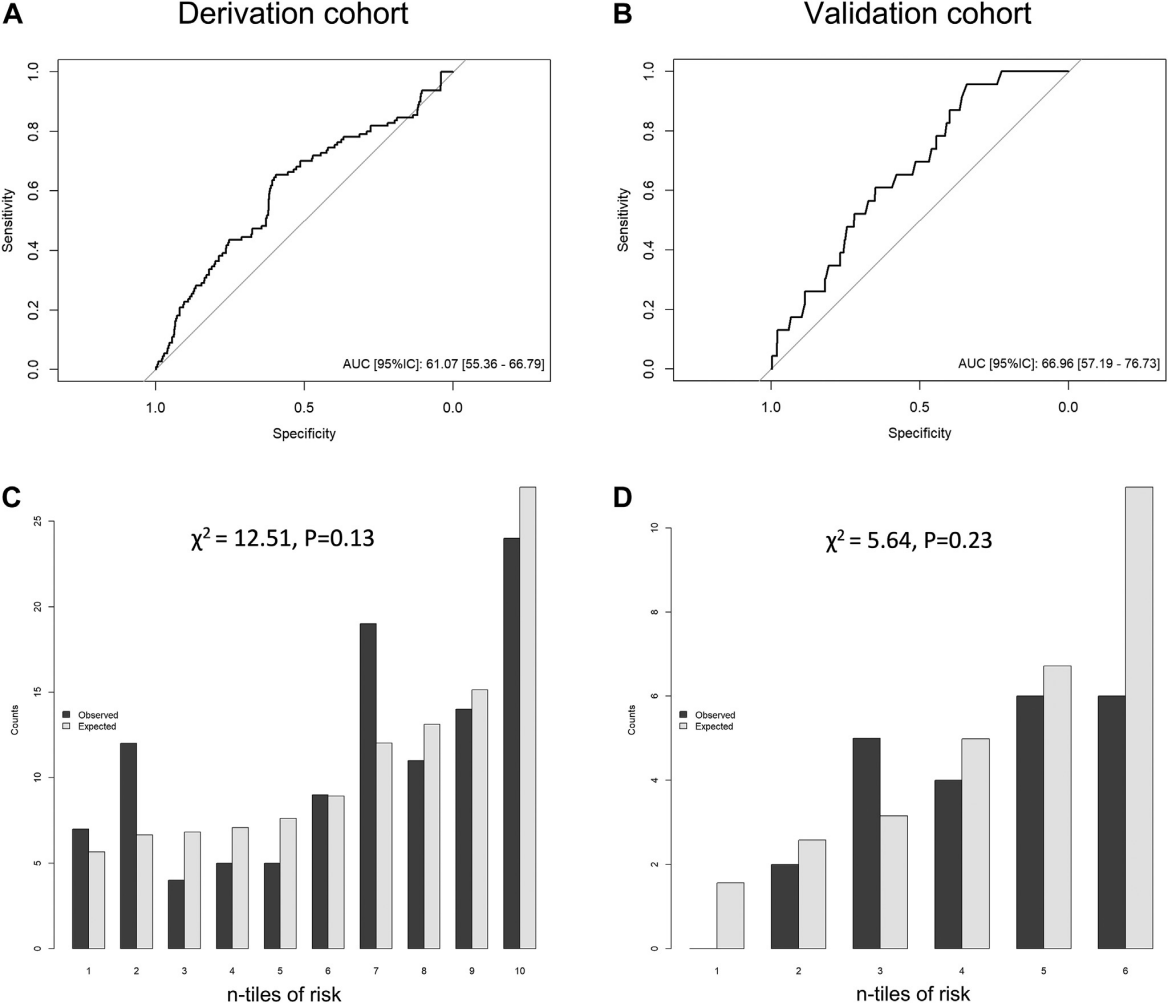
Discrimination capacity and calibration of the Atrial Fibrillation TRAck Complication risK (AF-TRACK) calculator. Receiving operator characteristics curves **(A, B)** and Hosmer-Lemeshow calibration tests **(C, D)** of the derivation and validation cohorts.

A logistic regression model was performed with a final dataset including 2943 individuals, 691 of whom underwent ≥2 procedures. A first model was fitted to assess the likelihood of undergoing repeat procedures and to provide an estimate of the ablation track length. Age (odds ratio [OR] 0.9 for each 10-year increase; *P* = .028), nonparoxysmal AF (OR 1.5; *P* <.001), and the center’s procedural volume (OR 4.4 for <100 ablations per year; *P* <.001) were independently associated with repeat intervention (Table 4). The final model (AF-TRACK calculator) was fitted to predict complications of undergoing an AF ablation track by adjusting by the propensity for repeat procedure. Age (OR 2.0; *P* = .006), female sex (OR 19; *P* = .002), heart failure (OR 2.2; *P* = .016), sleep apnea syndrome (OR 2.0; *P* = .014), and propensity for repeat ablation (OR 0.8; *P* <.009 for interaction with age) were associated with increased risk of complications (Table 4). There was no significant association between the inclusion period (OR 0.63 [0.38–1.05] for ablations performed in 2012 and after; *P* = .075), additional substrate ablation (OR 1.04 [0.64–1.71]; *P* = .86), or ablation technology (*P* = .242). The discrimination capacity of the AF-TRACK model was evaluated with receiver operating characteristic curve and showed an AUC of 0.61 [0.55–0.67] and good calibration (HL χ^2^ 12.5; *P* = .13) (Figure 1). Sensitivity analysis by center did not modify the performance of the model (AUC 0.55–0.61). Substrate ablation significantly interacted with propensity for repeat procedure (*P* = .003). This interaction was not included in the model because it failed to improve discrimination capacity and significantly diminished model calibration (*P* <.001 at H-L test).

**Table 4:** Logistic regression models to estimate propensity for repeat ablation and risk of complications

	Beta	SE	*P* value	OR	95% CI	
Propensity for repeat ablation						
(Intercept)	–2.612	0.160	.000	—	—	—
Age (per 10 y)	–0.099	0.042	.019	0.906	0.834	0.984
Nonparoxysmal AF	0.344	0.090	.000	1.411	1.182	1.685
Heart failure	–0.418	0.228	.067	0.658	0.421	1.029
Sleep apnea syndrome	0.277	0.152	.069	1.319	0.978	1.778
Center volume (≥100/y)	1.478	0.162	.000	4.386	3.194	6.022
Risk of complications of AF ablation track						
(Intercept)	–3.520	0.322	–	—	—	—
Propensity ≥2 ablations	–0.793	1.159	.494	0.452	0.047	4.384
Age (per 10 y)	0.693	0.254	.006	2.000	1.216	3.288
Female sex	0.636	0.208	.002	1.888	1.257	2.837
Heart failure	0.775	0.322	.016	2.170	1.155	4.077
Sleep apnea syndrome	0.677	0.277	.014	1.968	1.144	3.386
Propensity ≥2 ablations: Age (interaction)	–2.537	0.968	.009	0.079	0.012	0.527

AF = atrial fibrillation; CI = confidence interval; OR = odds ratio; SE = standard error.

The multivariate regression model identified heart failure (OR 2.6 [1.1–6.2]; *P* = .032) and significant valvular disease (OR 4.2 [1.2–13.9]; *P* = .021) as independent predictors of VAC. The prediction model showed acceptable discriminatory power with an AUC of 0.60 (0.52–0.69) after adjusting by the propensity for repeat procedure, with good calibration (HL χ^2^ 1.6; *P* = .814) (Supplementary Figures S1 and S2). (Detailed information on model development and results is available in Supplementary Tables S3 and S4, and Supplementary Figure S3.)

### External model validation

A single-center, independent cohort was used to assess the external validity of the model (N = 633). Description of the characteristics of the external population is given in Supplementary Table S5. The model outperformed when tested in the external cohort, showing adequate discrimination capacity (AUC 0.67 [0.57–0.77]) and calibration (HL χ^2^ 5.64; *P* = .23) (Figure 1). The prediction model for VAC was not validated because of the low number of vascular events in the external cohort (n = 4 [0.6%]).

### AF-TRACK calculator

**Figure 2:**
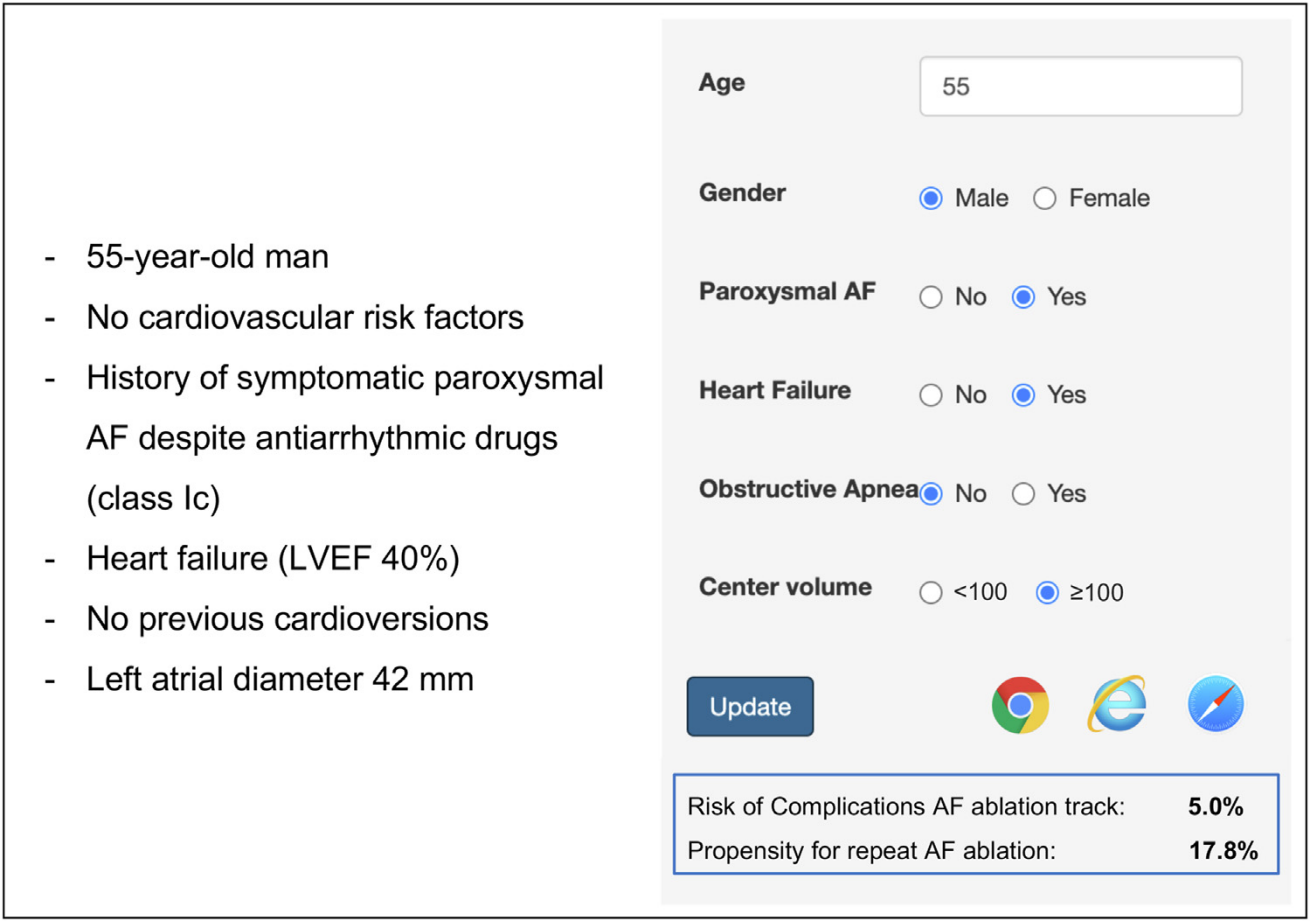
Case example of the Atrial Fibrillation TRAck Complication risK (AF-TRACK) calculator. Cumulative risk of complications and propensity for repeat procedure are provided. Details on the calculations for this example are given in the Supplementary Methods. AF = atrial fibrillation; LVEF = left ventricular ejection fraction.

The AF-TRACK calculator was developed as a simple, accessible, Web-based tool that allows easy access to risk stratification. The calculator is based on the validated AF-TRACK model to provide the overall cumulative risk of undergoing AAT, as well as detailed information on the probability of repeat procedure and risk of complications (Figure 2). This tool is accessible at https://aftrack.shinyapps.io/riskcalculator.

## Discussion

**Figure 3:**
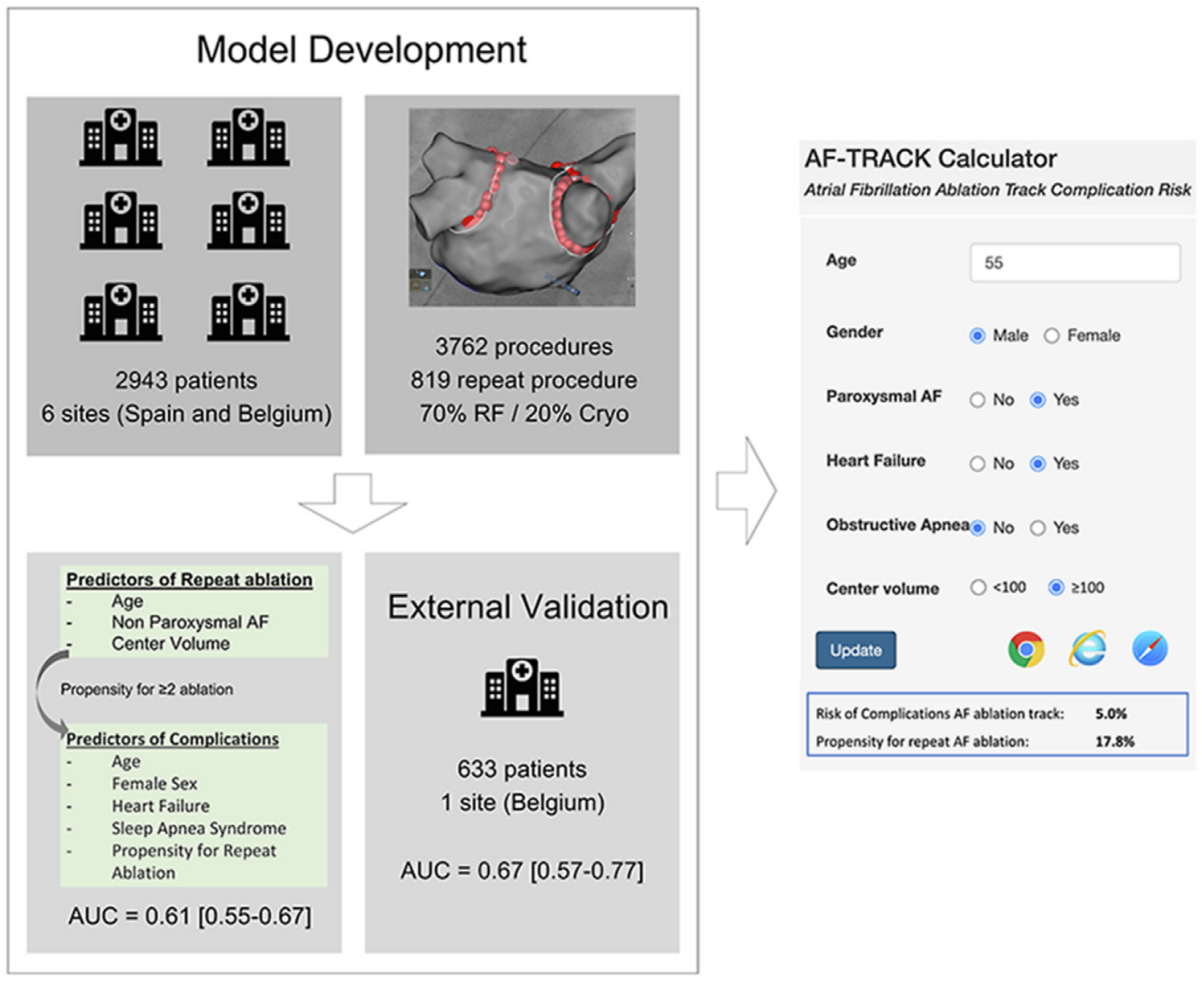
Summary of model development and validation. The Atrial Fibrillation TRAck Complication risK (AF-TRACK) calculator is an externally validated tool that provides individualized estimates of the risk of complications of the atrial fibrillation (AF) ablation track, based on the risk of procedural complications and the propensity for repeat ablation. AUC = area under the curve; Cryo = cryoballoon; RF = radiofrequency.

This study provides the first validated risk prediction model of major complications of AF ablation and allows for individualized preprocedural assessment through the Web-based AF-TRACK calculator. It also introduces the concept of AF ablation track as an invasive rhythm control strategy, which often involves >1 intervention to achieve rhythm and symptom control and thus implies a repetitive exposure to procedural risk (see Figure 3).

AF ablation is the cornerstone of AF treatment when medical treatment fails to provide rhythm control or symptom relief. The number of AF ablation procedures has increased dramatically in the last decade,^4^ accounting for almost 50% of all ablation procedures.^13^ As the evidence in favor of ablation over drugs increases, even as a first-line therapy,^14^^,^^15^ the number of AF ablation procedures is expected to keep rising in the future. Even though it is considered a safe procedure, AF ablation still is associated with a non-negligible 5%–6% risk of complications and up to 0.5% risk of early mortality.^6^^,^^16^ However, the inconsistency of the definition of complications across studies, as well as differences in operator experience and institutional protocols, preclude their comparability.^7^ As referenced in the 2017 consensus document, most data on complications come from high-volume centers; however, an increasing number of low-volume centers perform AF ablation (<50 procedures per year),^17^ and the rate of complications is expected to be higher than reported.^6^ Remarkably, clinical practice guidelines and consensus documents are merely descriptive and do not provide usable tools to facilitate the shared decision-making process based on an individualized risk of complications.^6^^,^^18^ The present international, multicenter study aimed to incorporate sites with high, medium, and low volume, in an effort to provide a wide spectrum of real-world clinical practice and an easy-to-use tool to estimate the risk of complications at an outpatient level.

**Figure 4:**
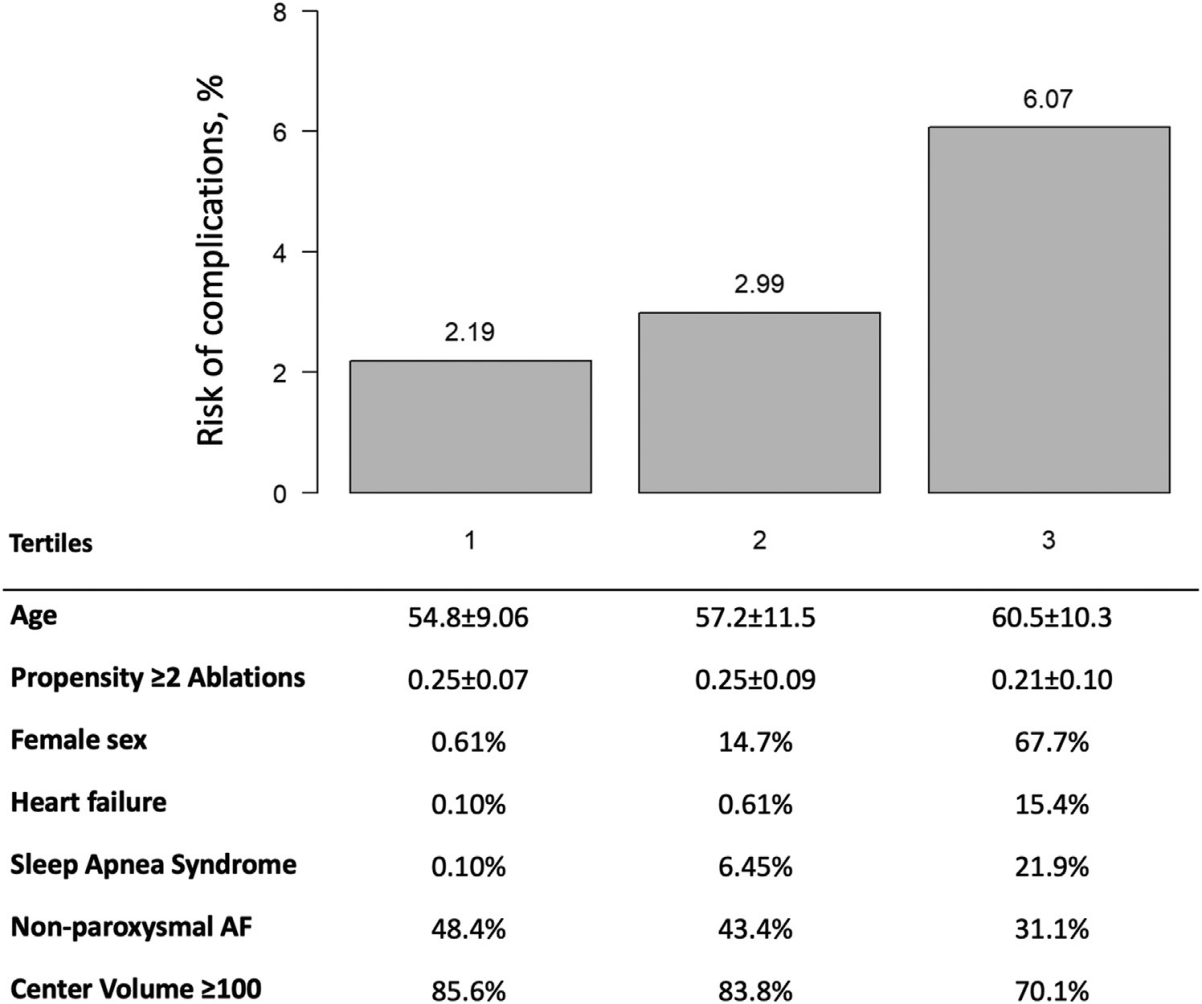
Prevalence of risk factors by risk tertiles. AF = atrial fibrillation.

Importantly, the safety of AF ablation is consistently reported as a procedure-related variable and does not consider the risk of repetitive exposure when more interventions are performed. A recent Swedish study reported that following a *de novo* AF ablation, the cumulative incidence of repeat procedure was ∼25% and ∼50% at 1 and 8 years, respectively.^4^ Repeat ablation is considered more effective than drugs following AF recurrence after de *novo ablation*,^19^ but caution must be taken because some evidence warns of a potential safety issue of repeat procedures.^20^ In order to address the influence of repeat procedures, our study incorporated into the model an estimate of the likelihood of repeat ablations, which allows for adjusting the overall cumulative risk of undergoing AAT. The predicted cumulative risk of undergoing AF ablation was found to be higher than previously reported, as the adjusted risk of repetitive exposure was not previously considered. Individual clinical characteristics are associated not only with increased risk of complications but also with higher probability of repeat procedure and, hence, increased cumulative risk estimates (Figure 4). Measures aiming to improve single-procedure success, thereby shortening the AAT length, are of utmost importance to reduce the overall cumulative risk of complications. The ability to create durable transmural lesions leading to persistent PVI is a major contributor to reducing repeat ablations and should be considered the first step in reducing the cumulative risk of AAT.

### Model development and validation

The purpose of the study was to provide a simple tool based on readily available clinical parameters that would allow easy risk assessment in the outpatient clinic. The addition of more sophisticated imaging parameters or biomarkers likely would improve the performance of the model but would dramatically reduce its clinical applicability. Consistent with previous reports, including recent United States registry data,^21^ age, female sex, clinical heart failure (but not LV ejection fraction), and sleep apnea syndrome were associated with increased risk of complications. Patients undergoing repeat procedures were found to be at 2-fold increased risk of complications compared to those receiving *de novo* ablation at univariate analysis. The effect of the propensity to undergo repeat ablation on the complication model has no direct interpretation, especially in the context of a strong interaction with age (inverse relationship). The propensity of repeat intervention was best predicted by age, heart failure, nonparoxysmal AF, and center’s volume (≥100 ablations per year). We acknowledge that procedural factors such as use of intracardiac echocardiography are key determinants of complication risk.^22^ Nonetheless, the main goal of the study was to develop a model based on the patient’s clinical, nonmodifiable factors to provide preprocedural risk assessment. Sensitivity analysis by center did not increase discrimination capacity.

To evaluate the applicability of the model to other populations, an internal and external validation was carried out. Remarkably, the AF-TRACK calculator showed better discrimination capacity when tested in an independent external cohort, providing evidence of the stability of the model.

Results from the predictive model for VAC should be interpreted given the relatively low number of VACs, the inability to externally validate the model, and the differences between the study cohort and current practice regarding ultrasound-guided puncture.

As with other interventions, AF ablation is associated with a non-negligible rate of complications. Accurate, individualized risk prediction is needed when balancing the pros and cons of undergoing this invasive treatment option. We advocate for an active, well-informed patient involvement in the decision process. The AF-TRACK calculator may provide clinicians and patients with additional valuable information to help in the decision-making process, but this must be viewed in the context of each center’s experience and institutional complication profile.

### Study limitations

Neither deaths nor atrioesophageal fistulas were documented in the derivation cohort. Some clinical characteristics, such as ethnicity or presence of chronic kidney/pulmonary diseases, were not available. Sleep apnea syndrome was not systematically screened. Catheter technology (ie, contact force sensing, cryoballoon generation) was not coded in most databases, and its association with complications could not be established. Vascular ultrasound currently is used in most AF ablation procedures, but it was not the standard of care during the data collection period. Therefore, the risk of vascular complications in the study population must be interpreted with caution if ultrasound-guided puncture is performed. Expanding the current dataset with external cohorts providing additional baseline clinical data and other complications (death/atrioesophageal fistula) may improve the performance and expand the applicability of the model. The model should be considered only in patients with characteristics similar to those of the study population and should be used as a complementary tool to adequately balance the risk-to-benefit ratio in the shared decision-making process.

## Conclusion

The proposed risk prediction model provides individualized estimates of the risk of major complications of the AF ablation track. The AF-TRACK calculator offers a validated, easy-to-use, Web-based clinical tool that allows clinicians and patients with AF to better calibrate the risk-to-benefit ratio of undertaking an invasive treatment strategy.

## Footnotes

Appendix Supplementary data associated with this article can be found in the online version at https://doi.org/10.1016/j.hroo.2022.07.013.
