# Supplementary material for: Personalized assessment of the cumulative complication risk of the atrial fibrillation ablation track: The AF-TRACK calculator
Source: Heart Rhythm O2. 2022 Aug 9;3(6Part A):656–64. doi: 10.1016/j.hroo.2022.07.013 (PMC9795263; doi:10.1016/j.hroo.2022.07.013)
Supplement: Supplemental Material [file mmc1.pdf]

SUPPLEMENTAL MATERIAL

Supplemental Methods

Case example from figure 2. Example on how the model computes the probability of having a complication.

55 years old man, with paroxysmal AF, with a previous diagnosis of heart failure and no history of significant valvular disease or sleep apnea syndrome scheduled for AF ablation in a center performing ≥100 procedures/years.

First, we compute the propensity of undergoing repeat ablation procedure -Prob(Abl>1)- as:

$$[1 + e^{-(-2.61183904-0.09857339*Agec/10 +0.34442118*NonPAF-0.41781667*HF+0.27672668*OSAS+1.47832611*Volume)}]^{-1}$$

For the example above:

$$[1 + e^{-(-2.61183904-0.09857339*(55-57.47)/10+0.34442118*0-0.41781667*1+0.27672668*0+1.47832611*1)}]^{-1} = 0.1784357$$

Finally, the probability of having a procedural complication, given the propensity of repeated ablation obtained in the first step (17.8%), is:

$$[1 + e^{-(-3.5200075-0.7932466*Prob[Abl>1]+0.6930246*Agec/10+0.6356663*Female+0.7747187*HF+0.6771413*OSAS-2.5373991*Prob[Abl>1]*Agec/10)}]^{-1}$$

And for the example above:

$$[1 + e^{-(-3.5200075-0.7932466*0.1784357+0.6930246*(55-57.47)/10+0.6356663*0+0.7747187*1+0.6771413*0-2.5373991*0.1784357*(55-57.47)/10)}]^{-1} = 0.04991751 = 5\%$$

Legends

- Sex: (0=male; 1=female)
- Age: centered age computed as age minus the average from the sample
- Non-PAF: Non-paroxysmal AF (0=paroxysmal AF; 1=persistent AF)
- HF: Heart failure (0=no; 1=yes)
- OSAS: sleep apnea syndrome (0=no; 1=yes)

Supplemental Results

Supplemental Tables

Table S1. Ablation set in first and repeat ablation procedures (full derivation cohort).

	1st Ablation	2nd Ablation	≥3 Ablations	Total	P Value
PVI	3044 (99.8%)	591 (81.2%)	70 (52.2%)	3705 (94.7%)	<0.001
Roof Line	431 (14.1%)	135 (18.5%)	35 (26.1%)	601 (15.4%)	<0.001
Posterior Box	56 (1.8%)	56 (7.7%)	30 (22.4%)	142 (3.6%)	<0.001
Mitral Line	250 (8.2%)	138 (19.0%)	65 (48.5%)	453 (11.6%)	<0.001
Anterior line	32 (1.0%)	43 (5.9%)	31 (23.1%)	106 (2.7%)	<0.001
CFAE	51 (1.7%)	31 (4.3%)	25 (18.7%)	107 (2.7%)	<0.001

Abbreviations. CFAE, complex fractionated atrial electrogram

Table S2. Patients' baseline characteristics according to number of procedures received.

	**1** *(N=2252)*	**≥2** *(N=691)*	**P value**
Age	58.7 [51.0;65.8]	57.0 [49.7;64.2]	0.004
Female	646 (28.7%)	164 (23.7%)	0.012
Hypertension	1031 (45.8%)	259 (37.5%)	<0.001
Diabetes mellitus	186 (8.26%)	43 (6.22%)	0.096
Body mass index	26.4 [23.6;29.1]	27.2 [24.3;29.7]	0.005
Non Paroxysmal AF	878 (39.0%)	331 (47.9%)	<0.001
Heart Failure	131 (5.82%)	26 (3.76%)	0.045
Coronary artery disease	120 (5.33%)	31 (4.49%)	0.436
Significant valvular disease	41 (1.82%)	7 (1.01%)	0.196
Sleep apnea syndrome	209 (9.28%)	70 (10.1%)	0.553
Left ventricular EF	60.0 [55.0;65.0]	60.0 [55.0;65.0]	0.706
CHA_2DS_2-VASc	1.00 [0.00;2.00]	1.00 [0.00;2.00]	<0.001
Left atrial diameter	42.0 [38.0;46.0]	43.0 [39.0;47.0]	0.005
Energy Source			<0.001
Radiofrequency	1597 (70.9%)	496 (71.8%)	
Cryoballoon	367 (16.3%)	53 (7.67%)	
Others	288 (12.8%)	142 (20.5%)	
Substrate ablation	368 (16.3%)	149 (21.6%)	0.002
Center volume (≥100)	1702 (75.6%)	645 (93.3%)	<0.001
Complications	69 (3.06%)	41 (5.9%)	0.001

Abbreviations. AF, atrial fibrillation; CHA_2DS_2-VA_2Sc, congestive heart failure, hypertension, age ≥ 75 years, diabetes mellitus, stroke or transient ischemic attack, vascular disease, age 65 to 74 years, sex category; EF, ejection fraction

Table S3. Patients' baseline characteristics by the presence of vascular access complications (derivation cohort)

	No VAC (N=2943)	VAC (N=46)	p value
≥2 ablation	691 (23.5%)	22 (47.8%)	<0.001
Age	58.3 [50.6;65.4]	61.5 [54.6;66.8]	0.072
Female sex	810 (27.5%)	17 (37.0%)	0.210
Hypertension	1290 (43.8%)	26 (56.5%)	0.116
Diabetes mellitus	229 (7.78%)	5 (10.9%)	0.583
Body mass index	26.6 [23.8;29.3]	26.6 [21.2;29.1]	0.671
Non-Paroxysmal AF	1209 (41.1%)	16 (34.8%)	0.477
Heart Failure	157 (5.33%)	6 (13.0%)	0.036
Coronary artery disease	151 (5.13%)	3 (6.52%)	0.732
Significant valvular disease	48 (1.63%)	3 (6.52%)	0.035
Sleep apnea syndrome	279 (9.48%)	4 (8.70%)	1.000
Left ventricular EF	60.0 [55.0;65.0]	60.0 [55.0;60.0]	0.356
CHA_2DS_2-VASc	1.00 [0.00;2.00]	2.00 [1.00;3.00]	0.005
Left atrial diameter	42.0 [38.0;46.0]	45.0 [40.0;49.5]	0.097
PVI-alone	2426 (82.4%)	42 (91.3%)	0.168
Center volume (≥100/year)	596 (20.3%)	8 (17.4%)	0.768

Table S4. Logistic regression model to estimate risk of vascular access complications

	UNIVARIATE				MULTIVARIATE			
	**OR**	**OR lower CI 95%**	**OR upper CI 95%**	**p-value**	**OR**	**OR lower CI 95%**	**OR upper CI 95%**	**p-value**
≥2 ablations	2.987	1.665	5.361	**<0.001**				
Propensity ≥2 ablation	0.663	0.025	17.529	0.806				
Age (per 10 years)	1.276	0.952	1.710	0.102	1.248	0.933	1.668	0.136
Female sex	1.544	0.844	2.824	0.159				
Hypertension	1.666	0.926	2.998	0.089				
Diabetes Mellitus	1.445	0.566	3.693	0.442				
Heart Failure	2.662	1.112	6.373	**0.028**	2.605	1.084	6.262	**0.032**
Coronary artery disease	1.290	0.396	4.206	0.673				
Significant valvular disease	4.208	1.262	14.036	**0.019**	4.147	1.234	13.929	**0.021**
Sleep apnea syndrome	0.909	0.324	2.555	0.857				
Center's volume (≥100)	1.206	0.560	2.599	0.632				
Cryoballoon (vs others)	0.572	0.204	1.604	0.288				
Period (≥2011)	0.860	0.462	1.601	0.634				

Table S5. Clinical characteristics and complications of the validation cohort.

	Total (N=652)	Complications (N=23)	No Complications (N=610)	p-value
**Clinical characteristics**				
Age	61.0 [54.0;68.0]	64.0 [60.0;69.5]	60.0 [53.0;68.0]	0.057
Female sex	221 (34.9%)	13 (56.5%)	208 (34.1%)	0.046
Arterial hypertension	135 (21.3%)	5 (21.7%)	130 (21.3%)	1.000
Heart failure	35 (5.53%)	4 (17.4%)	31 (5.08%)	0.033
Valvular disease	21 (3.32%)	1 (4.35%)	20 (3.28%)	0.546
Sleep apnea syndrome	54 (8.53%)	1 (4.35%)	53 (8.69%)	0.712
Paroxysmal AF	446 (70.5%)	12 (52.2%)	434 (71.1%)	0.085
**Complications**				
Tamponade	13 (2.05%)	13 (56.5%)	0 (0.00%)	<0.001
Stroke/TIA	1 (0.16%)	1 (4.35%)	0 (0.00%)	0.036
Symptomatic PV stenosis	0 (0.00%)	0 (0.00%)	0 (0.00%)	.
Phrenic nerve palsy	1 (0.16%)	1 (4.35%)	0 (0.00%)	0.036
Atrio-esophageal fistula	1 (0.16%)	1 (4.35%)	0 (0.00%)	0.036
Pulmonary edema	6 (0.95%)	6 (26.1%)	0 (0.00%)	<0.001
Death	2 (0.32%)	2 (8.70%)	0 (0.00%)	0.001

Abbreviations. AF, atrial fibrillation; TIA, transient ischemic attack; PV, pulmonary vein

Supplemental Figures

Figure S1. Receiving operator characteristic curve for vascular access complications predictive model.

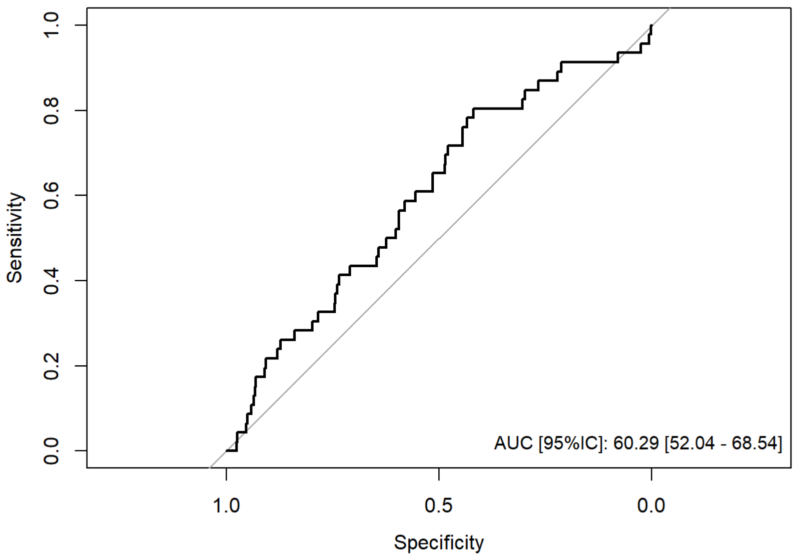

Figure S2. Hosmer-Lemeshow calibration test of the vascular access complications predictive model.

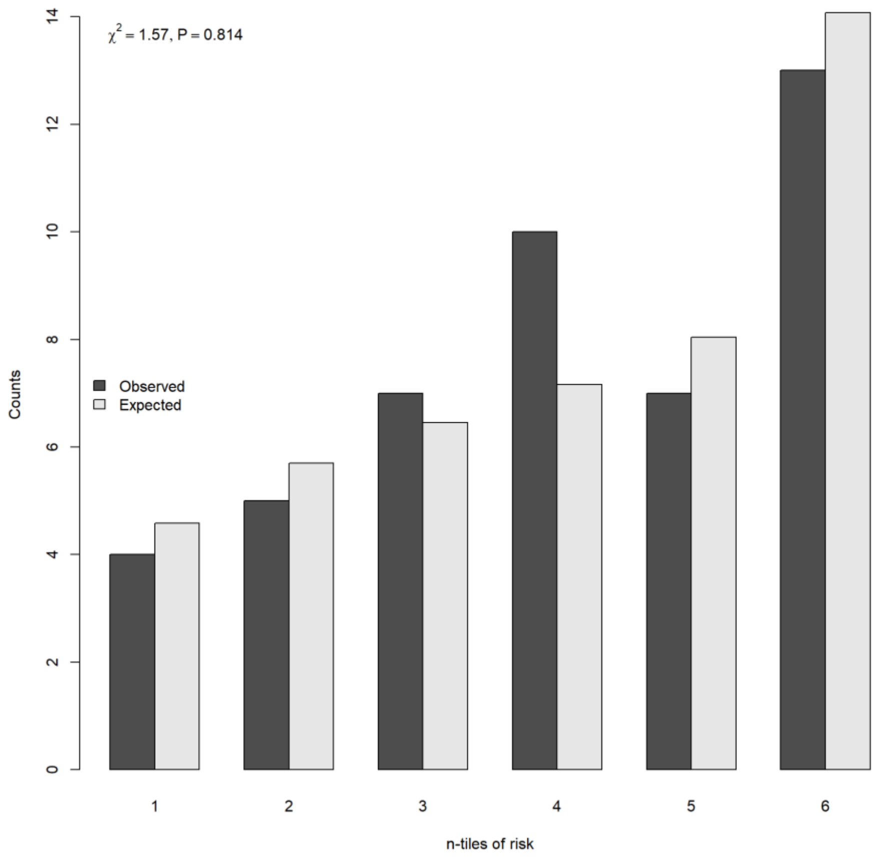

Figure S3. Prevalence of risk factors by risk tertiles for vascular access complications (VAC).

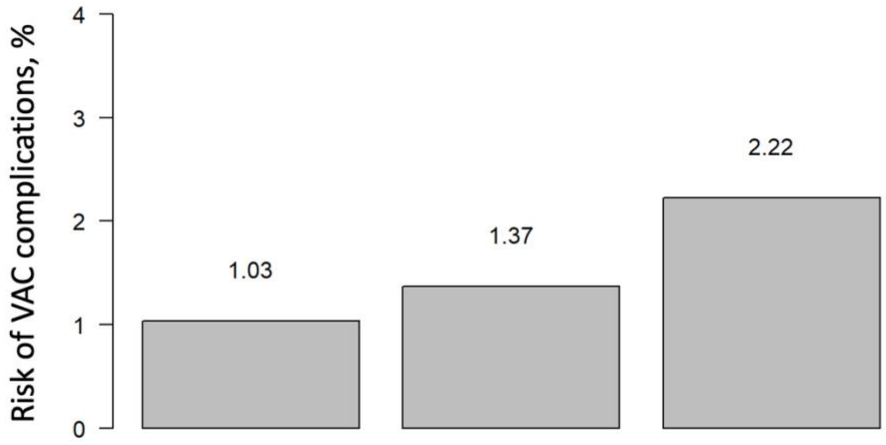

Tertiles	1	2	3
**Age**	46.1±6.70	59.3±2.85	67.2±7.45
**≥2 ablation**	0.26±0.09	0.24±0.08	0.21±0.09
**Heart failure**	0.00%	0.00%	16.4%
**Valvular disease**	0.00%	0.00%	5.13%
**Non-paroxysmal AF**	37.6%	40.6%	44.6%
